# Do organic substances act as a degradable binding matrix in calcium oxalate kidney stones?

**DOI:** 10.1186/s12894-021-00818-3

**Published:** 2021-03-26

**Authors:** Adi Adelman, Yaniv Shilo, Jonathan Modai, Dan Leibovici, Ishai Dror, Brian Berkowitz

**Affiliations:** 1 Department of Urology, Kaplan Medical Center, 7661041 Rehovot, Israel; 2 Department of Earth and Planetary Sciences, Weizmann Institute of Science, 7610001 Rehovot, Israel

**Keywords:** Chelating agents, Enzymes, Organic compounds, Chemolysis

## Abstract

**Background:**

Calcium oxalate (CaOx) stones are considered to be highly resistant to chemolysis. While significant organic matter has been identified within these stones, which is presumed to bind (inorganic) CaOx particles and aggregates, most chemolysis efforts have focused on methods to attack the CaOx components of a stone. We examine the feasibility of inducing chemolysis of CaOx kidney stones, within hours, by specifically attacking the organic matrix present in these stones.

**Methods:**

In contrast to previous studies, we focused on the possible “brick and mortar” stone configuration. We systematically tested, via in vitro experiments, the ability of an extensive range of 26 potential chemolysis agents to induce relatively fast disintegration (and/or dissolution) of a large set of natural CaOx stone fragments, extracted during endourological procedures, without regard to immediate clinical application. Each stone fragment was monitored for reduction in weight and other changes over 72 h.

**Results:**

We find that agents known to attack organic material have little, if any, effect on stone chemolysis. Similarly, protein and enzymatic agents, and oral additive medical treatments, have little immediate effect.

**Conclusions:**

These findings suggest that the organic and inorganic constituents present in CaOx stones are not structured as “brick and mortar” configurations in terms of inorganic and organic components.

## Background

The phenomenon of nephrolithiasis afflicts approximately 10% of the population globally, and its prevalence is rising continuously [1, 2]. Of the main types of kidney stones—calcium oxalate (CaOx), uric acid, struvite, hydroxyapatite, brushite and cystine—calcium-containing stones are by far the most prevalent, comprising as much as 80% of all stones.

Kidney stones have been the target for chemolysis for many years though with only limited success. Of the various kidney stones, CaOx stones in particular are considered to be the most resistant to chemolysis. Studies of oral intake treatments find little evidence of successful dissolution of existing CaOx stones (or, even of inhibiting stone formation [3–5]). In vivo chemolysis of kidney and bladder stones began seriously with the use of Renacidin, which appeared effective as a powerful dissolution agent for calcium phosphate, calcium carbonate, and magnesium ammonium phosphate (struvite) stones, but not for CaOx stones [6]; see also, e.g., [7] for calcium phosphate stones. If appropriate agents could be identified, the principal of in vivo chemolysis of CaOx kidney stones remains attractive [8, 9].

In efforts to investigate chemolytic behavior of CaOx stones, several in vitro studies have considered various potential agents. These agents have generally demonstrated poor efficacy in terms of percentage dissolved and relatively long times required for disintegration, for both real and synthetic stone types. Studies using artificial, *synthesized* “kidney stones”, consisting of relatively pure calcium oxalate aggregates and calcium phosphate crystals [9–11], yielded disappointing results of up to 10% stone weight loss over several hours, and 13–47% stone weight loss over days using enzymatic disintegration (oxalate decarboxylase and oxalate oxidase) to attack oxalate [10]. Moreover, in vitro studies that test chemolysis of *real* CaOx kidney stones harvested from human patients are similarly limited, particularly in terms of (1) the actual number of studies, and (2) the small number and type of chemolysis agents and stones tested in each study. For example, one report focused mostly on snake venom thrombin-like enzyme with the addition of antibiotics [12], while another [9] investigated natural and synthetic chelating reagents (citrate and EDTA) together with an antibiotic. These studies on real stones, too, demonstrated overall poor efficacy in terms of percentage disintegrated and/or time required for disintegration, e.g., ~ 10–50% disintegration by weight after 5 days [12], and up to ~ 10% over several (2–10) hours [9].

Significantly, though, with the partial exception of one investigation of an enzyme to specifically target proteins in the stone matrix [12], essentially all of these studies investigate agents that are expected to attack the CaOx (inorganic) components of a stone. And yet, a range of studies have identified significant organic matter, even as a matrix structure, within these stones, which is presumed to bind CaOx particles and aggregates [13–25]. Indeed, scanning electron microscope images (from these and other studies) show that CaOx stones are highly heterogeneous, containing layered, amorphous, and crystalline material. In this context, too, over 1000 proteins have been identified in kidney stones [20], which may form an organic protein matrix [13, 14]. However, while the literature mentions the *presence* of organic matter, it does not generally give *quantitative* measures of the relative amount of organic matter [13–21]; one series of studies yielded estimates of ~ 2.5% organic matrix by weight [22, 23]. Identification and quantification of *organic* constituents, whether from surface analysis or total sample analysis, remains difficult. Moreover, the precise nature and influence of organic matter remains uncertain, and the amount and type of organic matter varies among different stones, patient pathologies, and other factors. Given the assumption that the organic “matrix” in stones may act to bind inorganic components (such as CaOx particles), the overall stone structure may be that of a “brick and mortar” configuration, wherein the organic matter is the “mortar” and the inorganic constituents being the “bricks”; if so, then regardless of the specific stone configuration (e.g., containing layered, amorphous, and/or crystalline regions), it might be possible to achieve fast stone disintegration by attacking organic components.

In light of the highly limited information available on disintegration properties of real CaOx kidney stones, and to evaluate potential new routes for, ultimately, in vivo chemolytic treatments, the objectives of this study were to systematically analyze potential chemolysis agents on real kidney stones, effective within hours, and without regard to immediate clinical application. For the first time, we focus specifically on the question of whether CaOx stones can be treated as “brick and mortar” aggregates, which would enable chemolysis targeted to attack the organic material within CaOx stones [13–25]. We note that in all such studies, it is important to differentiate between “dissolution” and “disintegration”—while pure synthetic CaOx particles dissolve to various extents, CaOx kidney stones (which are aggregates of inorganic and organic materials) may also disintegrate into fragments when binding material is affected—so that the term “chemolysis” encompasses all processes of stone decomposition.

## Methods

### Collection and analysis of stones

Patients arriving to a medical institution for elective endourological procedures to remove kidney stones during March–May 2019 were invited to take part in this research. Written informed consent was obtained from all patient participants. Multiple stone *fragments* (hereafter referred to as “stones”)—as obtained from laser lithotripsy and percutaneous nephrolithotomy, without further treatment—removed during each endourological procedure were used in this research. The stone weights were in the range 10–165 mg (3–7 mm length along the major axis). One stone from each patient was analyzed to confirm that it was calcium-oxalate based (all containing mixtures (e.g., [16]) of monohydrate (> 70%) and dihydrate (< 30%) calcium oxalates), via x-ray diffraction. Overall, more than 100 stones collected from 15 patients were used.

### Chemolysis protocol

We surveyed, via in vitro experiments, a broad range of 26 potential chemolysis agents, divided into four groups (Table 1). These agents were selected specifically to target, in particular, organic binding material reported to be present in stone fragments, with the aim of achieving fast disintegration. The agents were chosen on the basis of existing medical literature and current clinical practice, folk remedies, and knowledge of known dissolution and digestion techniques of both inorganic and organic matter used in biogeochemical studies in the earth sciences.

**Table 1:** Selected potential chemolysis agents and concentrations

Type of chemolysis agent	Agent* (Supplier)	Concentration (Purity %)
Organic solvents	Toluene (Bio Lab, Israel)	Neat (99.7%)
	Ethanol (J.T. Baker, Holland)	Neat (99.5%)
	Acetone (Bio Lab, Israel)	Neat (99.8%)
	Acetonitrile (Bio Lab, Israel)	Neat (99.97%)
	Dimethylformamide, DMF (Merck)	Neat (99.8%)
	Tetrahydrofuran, THF (Sigma-Aldrich)	Neat (99.9%)
	Dimethylsulfoxide (Sigma-Aldrich)	Neat (99.5%)
Degradation of organic “cement” (binding agents)	H_2_O_2_ (Bio Lab, Israel)	8.8 M (30%)
	Copper nanoparticles + H_2_O_2_ (synthesized^26^, in house)	0.24 mM CuNP + 1.5% H_2_O_2_ (30%)
	Fenton: pH 2–4, Fe^2+^, H_2_O_2_ (synthesized^27^, in house)	1 mM Fe, 11 mM H_2_O_2_ (30%)
	Macerozyme R-10 plant digestion enzyme (Sigma-Aldrich)	20–40 enzyme units
	Drain cleaner (commercial, enzyme-based; HG, Israel)	As received
	Barbecue grill cleaner (commercial, enzyme-based; HG, Israel)	As received
Chelating agents and other agents	Potassium citrate, K_3_C_6_H_5_O_7_ · H_2_O (Sigma, USA)	16 mM (98%)
	EDTA (Sigma, USA)	0.1 M, 1 M, 2.5 M (99%)
	SDS (Ridel-de-Haen, Germany)	0.1 mM (90%)
	Triton X (Sigma, USA)	0.15 mM (> 99%)
	NH_4_OH (Merck, Germany)	Concentrated 16.7 M (25%)
Organic acids	Citric acid (Sigma-Aldrich)	0.1 M (99%)
	Ascorbic acid, vitamin C (Sigma, USA)	0.1 M (> 99%)
	Nicotinic acid (Sigma-Aldrich)	0.1 M (> 98%)
	Lactic acid (Sigma-Aldrich)	0.1 M (> 85%)
	Oxalic acid (Sigma-Aldrich)	0.3 M (> 85%)
	Acetic acid (Bio Lab, Israel)	0.1 M, concentrated 16.7 M (99.8%)
	Apple (cider) vinegar (commercial, food store)	5%
	Formic acid (J.T. Baker, Holland)	0.1 M, concentrated 21.7 M (98%)

^*^All agents were as received from manufacturers without further purification or modification

We emphasize that the solutions examined here are not generally appropriate for use in clinical applications; our intent here, before attempting to reach a translational approach at the clinical level, was to first understand whether focusing on the organic components of CaOx stones offers a potential avenue for further study. The justifications for these solution choices are as follows.

First, we investigated the potential dissolution of small organic molecules by strong organic solvents. These solvents might be expected to dissolve the cementing agent that is presumed to hold the inorganic debris (including CaOx particles which generally occur at sizes of up to about 20 microns) in the stone together [13–23]. If true, then stones might disintegrate into small (micron size) fragments that in turn could be flushed out.

The second group of chemolytic agents tested here were strong oxidation agents—copper nanoparticles with H_2_O_2_ [26] and Fenton reaction (pH 2–4, Fe^2+^, H_2_O_2_) [27]—both of which have been shown highly effective in degrading organic matter in complex matrices. These agents were applied to check their ability to degrade the organic substances and thus weaken or disintegrate the stone. Enzymatic mixtures to degrade organic matter—Macerozyme R-10 plant digestion pectinase enzyme [28], and even commercial drain cleaner and commercial barbecue grill cleaner—containing protease and other enzymes [10])—were similarly tested.

The third group we tested comprised potassium citrate, a chelator prescribed ubiquitously in clinical practice [3–5], EDTA, a chelating agent that was reported to effectively disintegrate CaOx [9, 11], anionic (SDS) and nonionic (Triton X) surfactants, and NH_4_OH. The underlying assumption in these cases was that strong chelators or surfactants could reverse chemical precipitation processes by shifting the equilibrium toward higher aqueous concentrations. In other words, it was expected that increasing the solution carrying capacity for calcium (and other metals) or particulate matter in solution would result in partitioning of calcium from CaOx to the solution and to stone disintegration. It was further assumed here that the strong chelating/complexing capacity of these compounds would replace or break existing bonding between organic substances and the CaOx (inorganic crystals), and in turn lead to stone chemolysis.

The fourth group comprised eight organic acids that can target a broad spectrum of inorganic and organic components. Organic acids also have chelating capabilities that can react with the CaOx. The rationale for testing this group is based on standard geochemical dissolution protocols applied for the disintegration or dissolution of stones and minerals, which have proven effective in other biogeochemical contexts [29, 30]. The organic acids were tested also because they include oft-suggested folk remedies via oral consumption, including citric acid, ascorbic acid (vitamin C), and acetic acid (particularly apple cider vinegar).

The experiments were performed in controlled conditions and in a comparative framework. Each stone was weighed and placed in a test tube containing a potential chemolysis agent solution at room temperature (24 °C), with some replicate tests performed with samples at 37 °C. In all cases, the same ratio of stone fraction mass to dissolving solution was employed; a relatively large relative volume of solution (1 mL of solution per 10 mg of stone) ensured a chemical excess of each potential chemolysis agent, and each sample was mixed periodically by gentle shaking. Note, too, that because stone *fragments* were tested, both the outer stone and internal surfaces were exposed to the solutions. Two replicates of stones (pairs of larger and smaller stones from the available set) with each potential agent in separate test tubes were tested. The samples were monitored for reduction in weight and any visual change. During the experiments, the stones were removed from solution, dried (by quickly removing excess solution with absorbent tissues), and weighed every hour for the first 3 h, and again after 12, 24, 48 and 72 h. Control experiments were performed in parallel, with several stones samples exposed in the same conditions to double deionized water.

Finally, for additional support and interpretation of the measurements, according to a standard geochemical method [29, 30], separate experiments were performed to determine stone dissolution properties by exposure to strong acids. In this context, HCl (8.8 M; Bio Lab, Israel, 37% purity) and HNO_3_ (11.1 M; Sigma-Aldrich, 70% purity) were used according to the same protocols as above to examine dissolution behavior. As a benchmark, similar experiments were also performed with pure, synthetic CaOx crystals (Sigma-Aldrich, > 99.9% purity). Synthetic kidney stones were not tested as they are known to be unrepresentative of real stones, particularly in terms of the binding material (e.g., [9–11]).

## Results

**Fig. 1:**
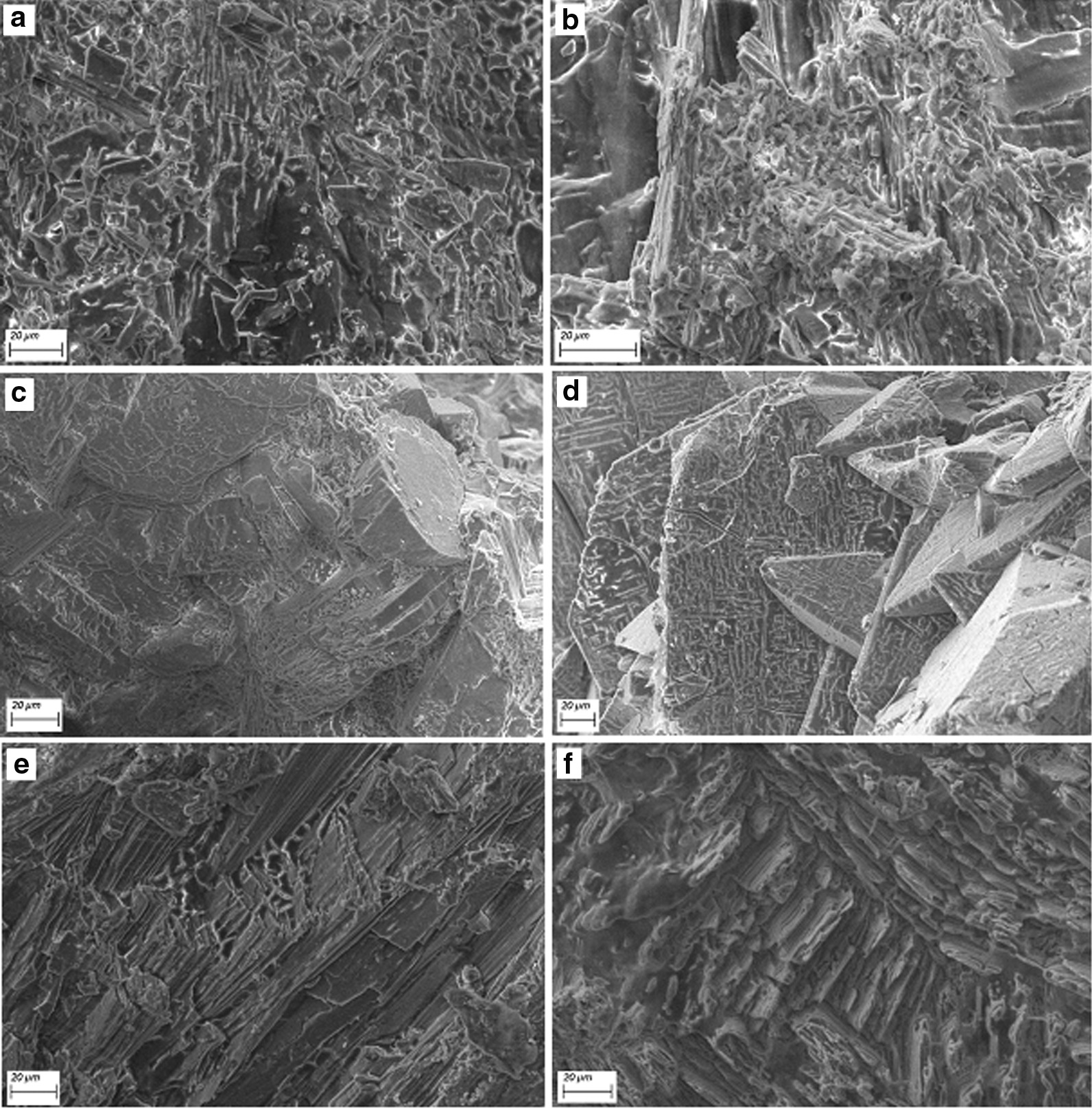
Representative SEM images of CaOx stone fragment (internal surfaces)—**a** before and **b** after, 24 °C, **c** before and **d** after, 37 °C—exposure to potential chemolysis agents, showing little change (similar images were found over a range of samples and at both temperatures); and **e** before and **f** after treatment at 37 °C with the potassium citrate solution, which displayed measured weight loss and chemolytic effects on the stone samples. Scale bars indicate 20 µm

We examined four groups of chemolysis agents (Table 1) on CaOx stones. It was found that none of these agents had any measurable impact (no change in weight) on stone disintegration, at least over the time frame of the experiments (72 h). Moreover, SEM analysis (Fig. 1) of some of these stone samples, before and after treatment, indicated essentially no significant modification of surface morphology. The only exception was the (oft-prescribed) agent potassium citrate, which indicated up to 30% disintegration (weight loss) of stone samples over this 72 h period; moreover, in addition to notable loss of stone mass, the remaining samples displayed changes in surface morphology, as seen from SEM images (Fig. 1e,f).

It is noted, too, that no change in the stones was observed in control experiments with double deionized water. Replicate tests performed with samples at 24 °C and at 37 °C showed that temperature had essentially no effect on the results.

We emphasize that concentrations of solutions were chosen to maximize possible chemolytic effects of each candidate agent. For illustration (Table 1), a potassium citrate at 16 mM is equivalent to 48 meq. This corresponds to a citrate concentration at least an order of magnitude higher than in urine [31]; to compare, oral potassium citrate therapy of 60–90 meq/d can increase urinary citrate by 30–115% [31].

## Discussion

Here, we assessed the potential of different solutions to dissolve or disintegrate CaOx stones within hours. In contrast to previous in vitro studies that focused on dissolution of (inorganic) CaOx components, we hypothesized—on the basis of numerous studies analyzing stone content and structure—that the organic matrix in the stones [13–23] may lead to a “brick and mortar” configuration, and thus serve as an “Achilles heel”, enabling fast stone disintegration by attacking organic components. We stress again that we use the term “brick and mortar” to differentiate between the two kinds of components—inorganic and organic—which in some embodiments may appear as distinct regions or layers of calcium oxalates and organic substances. In this context, we considered four groups of solutions to examine different possible modes of chemolytic processes of kidney stones that can attack (principally) organic constituents (see Table 1 and Methods above for the rationale of testing these agents).

**Fig. 2:**
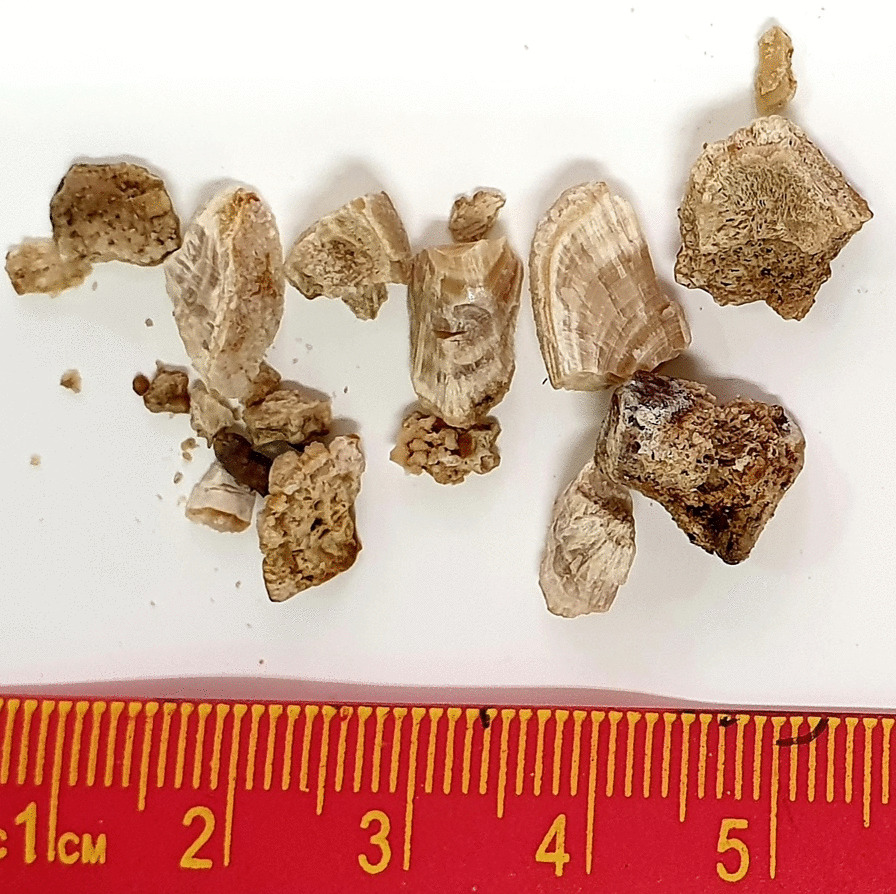
Example of CaOx stone fragments from a single patient. Note the various external and internal views of the stone fragments, some showing regions with visible internal layering, and others showing more heterogeneous or amorphous internal structures

We argue that the lack of change in stone weight—essentially no dissolution and certainly no fragmentation/disintegration of the stone fragments—is a clear indication that the stones do not consist of a “brick and mortar” configuration. Given the usual structure of kidney stone fragments, which contain layered materials and amorphous aggregates of CaOx crystals and organic matter (see Fig. 2), it is therefore deeply significant that none of the agents known to attack organic matter by direct chemolysis and/or chelation—organic solvents, degraders of organic binding material, chelating agents—had any noticeable effect on disintegration (or dissolution) of the stones. Moreover, none of the folk remedies had noticeable impact, at least at the concentrations we tested. As a consequence, it appears infeasible to quickly disintegrate stones by attempting to attack, specifically, the organic material in the stones.

We attribute these results to the recognition that a distinct organic matrix is not present, so that organic and inorganic constituents are not structured as “brick and mortar” configurations. Rather, it appears that real CaOx stone structures involve aggregates and inclusions that interweave inorganic and organic constituents having a wide range of (sub)crystal structures. This conclusion is further supported by analysis of dissolution properties by mineral acids that target a broad spectrum of both organic and inorganic materials: parallel tests using the above protocols with concentrated HCl (8.8 M) and HNO_3_ (11.1 M) yielded up to 100% dissolution within 0.5–3 h, with no detectable (visually, and following filtration on Whatman GF/A (Sigma-Aldrich) filters) particulate matter in solution. In contrast, similar tests using pure synthetic CaOx crystals show dissolution rates at least 1–2 orders of magnitude faster than human CaOx stones. The same acids at lower concentration—0.1 M—showed negligible dissolution even after 72 h; for comparison, HCl at 0.1 M is similar to that of gastric acid (100 meq). Detailed comparisons showing such behavior, between synthetic CaOx crystals and powdered real CaOx stones, appear elsewhere [32]. Thus, it appears that only strong agents that can simultaneously attack inorganic and organic components lead to fast stone dissolution.

We emphasize here that the findings from this systematic survey do not rule out the possibility (1) that some agents, such as potassium citrate or EDTA, may have a softening or slow dissolution effect on existing CaOx kidney stones, over longer time periods, and/or (2) that some of these agents, as well as others, may be beneficial in inhibiting or preventing formation of entirely new stones. [We note parenthetically, too, but in a different context, that natural, in vivo dissolution may occur over thin, nanometer layers of CaOx stones, with estimated time scales of hours [18].]

A unique feature of this study—systematic analysis of the effects of a wide range of potential dissolution agents on actual CaOx kidney stones—has an inherent limitation, namely, the lack of uniform stone size, structure, and composition among real stone samples even from the same patient. On the other hand, synthetically-grown kidney stones are relatively homogeneous, and in particular do not contain the potentially critical impurities, including organic matter, present in real stones. To reduce the uncertainty and variability in our survey, stone fragments from the same patient were tested where possible, in parallel. We also mediated the size differences by keeping a constant ratio of solution to stone mass. We emphasize, too, that stone *fragments* were employed, so that possible dissolution—a surface phenomenon—was maximized by solution exposure to both internal and external portions of each stone. In spite of the inherent variability in surface area, and use of replicates, similar behavior (essentially no dissolution or disintegration) was found among treatments.

## Conclusions

Our study is the first to examine the feasibility of inducing chemolysis of CaOx kidney stones, within hours, by specifically attacking the organic matrix present in CaOx stones. This matrix is often reported to be a possible binding agent between CaOx particles. In contrast to previous studies, we focused on the possible “brick and mortar” stone configuration, in an effort to gain basic insight into disintegration behavior of these stones. We systematically tested, via in vitro experiments, the ability of an extensive range of 26 potential chemolysis agents to induce relatively fast disintegration (and/or dissolution) of a large set of natural CaOx stone fragments, extracted during endourological procedures, without regard to immediate clinical application.

Within time periods of up to 72 h, we find—notably—that a broad range of biogeochemical agents known to attack organic material have little, if any, effect on the stones. Similarly, protein and enzymatic agents, and oral additive medical treatments (primarily potassium citrate pills and lemon juice), have little effect. These findings suggest that the organic and inorganic constituents present in CaOx stones are not structured as “brick and mortar” configurations, so that stone disintegration does not occur readily by attacking organic components. Rather, CaOx stones represent more complex aggregates that interweave inorganic and organic constituents, with wide ranges of (sub)crystal structures and inclusions.

To reach a translational approach at the clinical level, one must first understand the disintegration behavior of kidney stones and potential/perceived prospects for fast, in vivo disintegration. It remains to evaluate new routes for fast in vitro breakdown, and ultimately, in vivo, CaOx chemolytic treatments.

## Data Availability

All data appear in paper.

## Abbreviations

CaOx: Calcium oxalate
